# The Discovery of Dabigatran Etexilate

**DOI:** 10.3389/fphar.2013.00012

**Published:** 2013-02-12

**Authors:** Joanne van Ryn, Ashley Goss, Norbert Hauel, Wolfgang Wienen, Henning Priepke, Herbert Nar, Andreas Clemens

**Affiliations:** ^1^Department of CardioMetabolic Disease Research, Boehringer Ingelheim Pharma GmbH & Co. KGBiberach an der Riss, Baden-Württemberg, Germany; ^2^Department of CardioMetabolic Disease Research, Boehringer Ingelheim Pharmaceuticals Inc.Ridgefield, CT, USA; ^3^Department of Medicinal Chemistry, Boehringer Ingelheim Pharma GmbH & Co. KGBiberach an der Riss, Baden-Württemberg, Germany; ^4^Department of Respiratory Diseases Research, Boehringer Ingelheim Pharma GmbH & Co. KGBiberach an der Riss, Baden-Württemberg, Germany; ^5^Department of Lead Identification and Optimization Support, Boehringer Ingelheim Pharma GmbH & Co. KGBiberach an der Riss, Baden-Württemberg, Germany; ^6^Global Clinical Development and Medical Affairs, Boehringer Ingelheim Pharma GmbH & Co. KGIngelheim, Baden-Württemberg, Germany

**Keywords:** dabigatran, thrombin, oral anticoagulant, warfarin, stroke, atrial fibrillation, venous thromboembolism

## Abstract

Thromboembolic disease is a major cause of mortality and morbidity in the developed world and is caused by an excessive stimulation of coagulation. Thrombin is a key serine protease in the coagulation cascade and numerous efforts have been made to develop safe and effective orally active direct thrombin inhibitors (DTIs). Current anticoagulant therapy includes the use of indirect thrombin inhibitors (e.g., heparins, low-molecular-weight-heparins) and vitamin K antagonists such as warfarin. However there are several caveats in the clinical use of these agents including narrow therapeutic window, parenteral delivery, and food- and drug–drug interactions. Dabigatran is a synthetic, reversible DTI with high affinity and specificity for its target binding both free and clot-bound thrombin, and offers a favorable pharmacokinetic profile. Large randomized clinical trials have demonstrated that dabigatran provides comparable or superior thromboprophylaxis in multiple thromboembolic disease indications compared to standard of care. This minireview will highlight the discovery and development of dabigatran, the first in a class of new oral anticoagulant agents to be licensed worldwide for the prevention of thromboembolism in the setting of orthopedic surgery and stroke prevent in atrial fibrillation.

## Introduction

**Figure 1:**
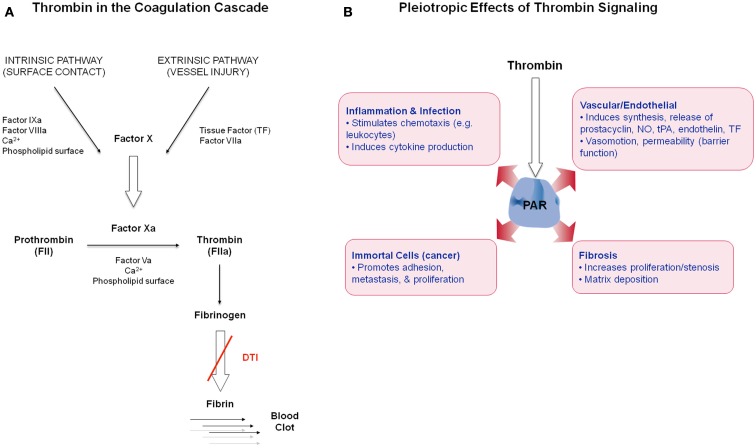
**(A)** Simplified schematic of thrombin in the coagulation cascade. **(B)** Overview of thrombin’s non-hemostatic effects via PAR receptor signaling in diverse cell types.

Thrombin is a serine protease and is the main effector protease in the blood coagulation cascade (Figure 1A), exhibiting both pro- and anticoagulant properties (Griffin, 1995; Di Cera, 2008). Thrombin (FIIa) is generated via proteolytic cleavage from inactive prothrombin (FII) by factor Xa (FXa) in the prothrombinase complex, which assembles when circulating coagulation factors come into contact with tissue factor (TF) on exposed extravascular tissues. Thrombin plays a central role in the initiation and propagation of thrombotic disease by activating platelets, catalyzing fibrinogen conversion into fibrin, and promoting clot stabilization (Lane et al., 2005). Thrombin activates upstream factors in the cascade to amplify the coagulation response and enhance thrombin generation. Its activity is inhibited via endogenous circulating anticoagulants including antithrombin (AT), heparin cofactor II (HCII), and binding to the cofactor thrombomodulin (TM) to activate the anticoagulant protein C (Griffin, 1995). Thrombin also induces many cellular effects via a family of G-protein coupled protease activated receptors (PAR), PAR1, PAR3, and PAR4 (Coughlin, 2005; Figure 1B). The expression of PAR1 on the endothelium and vasculature, in both physiological and disease states, suggests that thrombin plays a role in these contexts.

Established anticoagulation therapy has targeted thrombin. The parenteral agents heparin and low molecular weight heparins (LMWH) catalyze the inhibition of thrombin by AT and are used for prophylaxis and treatment of venous thromboembolism (VTE) in many indications (Hirsh et al., 2008). Other approved parenteral thrombin inhibitors include the recombinant hirudin lepirudin and synthetic direct thrombin inhibitor (DTI) argatroban (Coppens et al., 2012), for predominantly heparin-induced thrombocytopenia (Yeh and Jang, 2006), and the oligopeptide bivalirudin in percutaneous coronary interventions (Warkentin et al., 2008).

As the only available oral therapy, warfarin and its derivatives have been used for over 50 years in long-term anticoagulation treatment (Hirsh et al., 1998). Warfarin, a Vitamin K antagonist (VKA), blocks the biosynthesis of thrombin and other coagulation factors and has proven effective in inhibiting thrombosis. However, administration is problematic due to its slow onset-offset of activity, and drug–drug and drug-food interactions which require frequent monitoring and dose adjustment to maintain appropriate anticoagulation (Mann, 2005).

Research activities in many pharmaceutical companies have focused on the identification of novel thrombin inhibitors. In 2004 a new DTI ximelagatran (AstraZeneca; Gustafsson et al., 2004) gained approval in several countries for short-term anticoagulation treatment after orthopedic surgery, but was later withdrawn due to hepatotoxicity reports. Other companies concentrated their research activities on discovering FXa inhibitors (Pinto et al., 2010) including two recently approved FXa inhibitors – rivaroxaban and apixaban for treatment and prevention of VTE after orthopedic surgery (Pinto et al., 2007; Perzborn et al., 2011) and stroke prevention in atrial fibrillation (Granger et al., 2011; Patel et al., 2011). Two additional FXa inhibitors, edoxaban (Furugohri et al., 2008) and betrixaban (Zhang et al., 2009), are completing Phase III trials.

This review will focus on the development of dabigatran (Hauel et al., 2002), the first new oral anticoagulant (NOAC) to gain market approval for long-term indications 50 years after introduction of warfarin. Dabigatran is approved in over 70 countries, including the U.S., Canada, Europe, and Japan, for stroke prevention in patients with atrial fibrillation and for the prevention of thrombosis after orthopedic hip and knee surgery. Dabigatran is a potent, reversible, and direct inhibitor of thrombin, with a good efficacy, safety, and tolerability profile as compared to both warfarin and LMWH (Hankey and Eikelboom, 2011).

## Dabigatran Discovery and Preclinical Pharmacology

Twenty years ago an X-ray crystal structure of a bovine thrombin complex formed with the peptide-like benzamidine based inhibitor NAPAP revealed the conformation of an enzyme-bound thrombin inhibitor and its interactions with the residues of the active site cleft (Brandstetter et al., 1992). This data spurred the design of a new class of inhibitor molecules with increased inhibitory potency toward human α-thrombin and acceptable metabolic stability and pharmacokinetic properties (Davis and Teague, 1999; Hauel et al., 2002).

**Figure 2:**
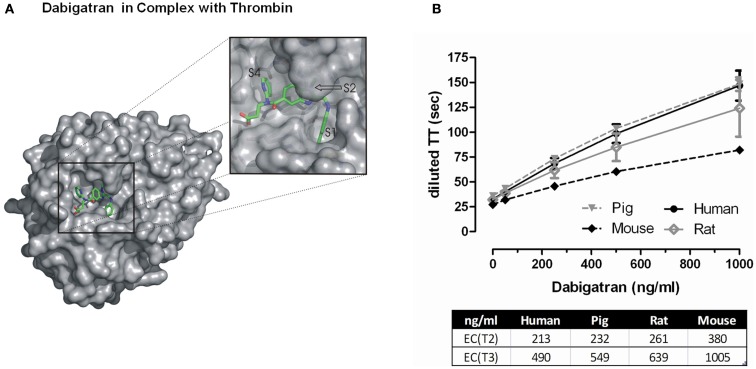
**(A)** Surface representation of FIIa bound to dabigatran. The insert shows a zoom into the active site cleft of the enzyme. The most prominent feature of the ligand-protein interaction interface is the deep S1 pocket in which the benzamidine moiety binds. The 60-loop insertion of FIIa with the prominent Trp-60D occludes a hydrophobic S2 pocket in which the methylbenzimidazole of dabigatran nicely fits. The S4 pocket is rather shallow pocket that prefers to bind aromatic moieties of inhibitors. Dabigatran occupies the S4 pocket with its pyridyl ring that forms an edge-on CH…π interaction with Trp-215 at the floor of the pocket and places its propionic acid group into the solvent exposed S3 pocket. **(B)** Effect of increasing concentrations of dabigatran on diluted thrombin time measurements in different species. The EC(*T*2) and EC(*T*3) represent the effective concentration of dabigatran to prolong the clotting time either twofold or threefold. Data represented as mean ± SE, *n* = 4–5.

A lead compound (dabigatran; Figure 2A) was identified because of its favorable selectivity profile and strong *in vitro* and *in vivo* activity, exhibiting long anticoagulation duration in rats after i.v. administration and toleration at high doses (Wienen et al., 2007a). However it was not orally active due to its polarity and the compound was converted into an orally active prodrug (dabigatran etexilate; Himmelsbach et al., 1995). Given orally to rhesus monkeys, this prodrug exhibited strong and long lasting anticoagulant effects as measured by the activated partial thromboplastin time (aPTT) *ex vivo* (Wienen et al., 2007a). Based on its promising profile, dabigatran etexilate was selected for clinical development.

### Molecular mechanism of action of dabigatran

Dabigatran inhibits human thrombin in a concentration-dependent and competitive fashion, with a *K_i_* of 4.5 nM. This inhibition is rapid and reversible, and comparison to IC_50_ values for other coagulation proteases demonstrated its high selectivity for thrombin (Wienen et al., 2007a). Dabigatran inhibits both clot-bound and free thrombin, and this binding is independent of whether thrombin is bound via the exosite to fibrin or is present as free enzyme in plasma (van Ryn et al., 2008).

Thrombin generated on the platelet surface is a potent agonist mediating platelet activation. Dabigatran inhibits thrombin-induced platelet aggregation, but has no inhibitory effect on platelet aggregation induced by arachidonic acid, collagen, or ADP (Wienen et al., 2007a). Dabigatran also effectively inhibits TF-induced thrombin generation in human platelet poor plasma (PPP) in a concentration-dependent manner.

### *In vitro* antihemostatic effects of dabigatran

Consistent and potent *in vitro* anticoagulant activity of dabigatran has been demonstrated using clotting assays across several species (Wienen et al., 2007a). A doubling of the aPTT, prothrombin time (PT), and ecarin clotting time (ECT) is observed at dabigatran concentrations ranging from 0.1 up to 4.6 μM, with the ECT being the most sensitive parameter for anticoagulant activity. The thrombin time (TT) assay is extremely sensitive to dabigatran’s effects and the commercially available Hemoclot^®^ Thrombin Inhibitor assay (Hyphen BioMed, Neuville-sur-Oise, France) is a diluted thrombin time (dTT) assay sensitive enough for accurate quantitative measurement of dabigatran activity across a broad concentration range (van Ryn et al., 2010). Thrombin inhibition by dabigatran was comparable in pig and human plasma, and inhibition of rat thrombin was ∼20% less potent than human, and mouse thrombin ∼twofold less potent than human (Figure 2B).

### *Ex vivo* antihemostatic effects of dabigatran

Significant dose- and time-dependent anticoagulant efficacy *ex vivo* has been demonstrated after i.v. administration of dabigatran to rats and rhesus monkeys. In rats, doses of 0.3, 1, and 3 mg/kg i.v. produce a maximum prolongation of the *ex vivo* aPTT to 29, 159, and 582 s, respectively, 5 min after administration. In rhesus monkeys, i.v. administration (0.15, 0.3, or 0.6 mg/kg) of dabigatran prolongs the aPTT to 47.3, 70.1, and 98.9 s, respectively, 5 min after administration and this is sustained beyond 8 h. Notably, single oral doses of 1, 2.5, and 5 mg/kg administered to conscious rhesus monkeys all revealed a significant and long lasting (>8 h) prolongation of the aPTT (Hauel et al., 2002; Wienen et al., 2007a).

### Venous and arterial antithrombotic effects of dabigatran

Infusion of dabigatran in a rat model of induced venous thrombosis inhibited clot formation dose-dependently and completely (Wienen et al., 2007b). No significant increase in bleeding time was observed at the maximum effective antithrombotic dose. In the same model, dabigatran etexilate administered orally between 0.5 and 7 h prior to thrombus induction resulted in a dose and time-dependent inhibition of thrombus formation. In a rabbit arterio-venous shunt model of thrombosis, infusion and oral administration of dabigatran revealed a dose-dependent inhibition of clot formation (Wienen et al., 2007c). In both the rat and rabbit thrombosis models the observed antithrombotic effects were inversely correlated with a dose and time-dependent prolongation of the *ex vivo* aPTT.

In the Folts model of arterial thrombosis in pigs, aspirin (ASA) treatment reduced cyclic flow reductions by 40% and dabigatran reduced by 44%. Given together, the two compounds reduced closures by 88% and proved to be safe and effective in this model. Additionally, a complete closure of the injured vessels was not observed at clinically relevant plasma levels in the dabigatran-treated and the dabigatran-ASA treated animals, demonstrating effectiveness in an arterial thrombosis setting (van Ryn et al., 2009).

## Pleiotropic Effects of Thrombin – Preclinical Observations

In addition to its role in hemostasis, thrombin directly and indirectly affects cell behavior and responses in a variety of tissue types via PAR receptor signaling, and has been implicated in a variety of diseases (Coughlin, 2005), suggesting other potential indications where thrombin inhibition could be beneficial. These therapeutic areas include diseases underscored by inflammation (van der Poll et al., 2011), infection (Levi et al., 2012), fibrosis (Chambers and Laurent, 2002), and cancer (Palumbo and Degen, 2000), among others. To date, many disease areas where thrombin-PAR signaling has been implicated are being investigated preclinically for beneficial effects of direct thrombin inhibition by dabigatran and several are described below. However effects in the clinic are unknown.

### Atherosclerosis

There is increasing evidence in experimental atherosclerosis that inflammation and coagulation play an important role in atherosclerotic development (Borissoff et al., 2009, 2011). In separate studies utilizing the Apo E^−/−^ mouse model of atherosclerosis, mice were fed high fat control diet or diet supplemented with dabigatran for up to 20 weeks. In all studies, dabigatran-fed mice exhibited reduced atherosclerotic lesion size along with enhanced plaque stability, improved endothelial function, and reduced oxidative stress (Preusch et al., 2010; Kadoglou et al., 2012; Lee et al., 2012). Another study in a procoagulant/atherosclerotic mouse model (ApoE^−/−^:TM^pro/pro^) having elevated coagulation activity due to a loss-of-function mutation in TM demonstrated that dabigatran significantly reduced plaque area and stenosis after a carotid cuff injury (Borissoff et al., 2010).

### Cancer

A link between cancer and thrombosis has long been recognized (Iodice et al., 2008) and there is experimental evidence implicating PAR1-mediated events and expression with tumor growth and metastasis (Booden et al., 2004). Studies testing the effects of dabigatran treatment (4 weeks) in a syngeneic mouse model of breast cancer demonstrated a reduction in both primary tumor growth and metastasis in dabigatran-treated mice as compared to control (DeFeo et al., 2010).

### Fibrosis

One hallmark of atrial fibrillation and many other degenerative diseases (e.g., renal disease, idiopathic pulmonary fibrosis) is the extent of fibrosis (Tan and Zimetbaum, 2011). Activation of coagulation following tissue injury is an early fibrotic response leading to the deposition of fibrin. Notably, thrombin activation of PAR1 can differentiate normal lung fibroblasts into a myofibroblast phenotype, inducing fibrogenic cytokine release and increased expression of extracellular matrix proteins (Bogatkevich and Silver, 2009). These effects are inhibited *in vitro* by dabigatran, and in bleomycin-induced pulmonary injury in mice chronic dabigatran etexilate treatment reduced the extent of fibrosis and collagen release (Bogatkevich et al., 2011).

### Infections with *Staphylococcus aureus*

The potential of *S. aureus* to coagulate blood is used as a diagnostic feature of the pathogen and more recently it has been shown that *S. aureus* achieves this by secreting a procoagulant, called coagulase (McAdow et al., 2011). Coagulase directly converts prothrombin into thrombin, bypassing upstream coagulation and allows *S. aureus* to envelope itself in protective fibrin strands, which may aid in its resilience to antibiotics (Vanassche et al., 2011). Dabigatran directly inhibits coagulase, unlike heparin and hirudin, and thus prevents fibrin formation and may increase the sensitivity of *S. aureus* to antibiotics (Vanassche et al., 2010, 2011, 2012).

## Clinical Studies and Indications

For 50 years VKAs have been the standard in chronic anticoagulation treatment, however their many associated drawbacks has led to significant clinical underuse in atrial fibrillation patients. There is a significant unmet medical need to develop anticoagulation treatment that is easy to use, predictable, effective, and safe. Dabigatran etexilate represents the first broadly approved NOAC with these attributes.

Dabigatran has a fast onset of action (peak plasma concentrations 2–3 h after ingestion) with a half-life of 12–14 h (Stangier and Clemens, 2009). It does not require routine coagulation monitoring, has no drug-food interactions, is not metabolized by cytochrome P 450 enzymes, and does not require dose adjustment for moderate liver disease (Garnock-Jones, 2011). As a substrate for the Pgp transporter there is the potential for drug interactions with agents using this transporter (e.g., verapamil, amiodarone, rifampicin), which may necessitate dose adaptation (Huisman et al., 2012; Härtter et al., 2012). Dabigatran is 80% renally excreted, thus prior to commencing dabigatran treatment renal function should be assessed, and in the instance of emergency situations dabigatran is dialyzable due to low protein binding (∼30%; Huisman et al., 2012). As with all anticoagulants, dabigatran’s most common side effect is bleeding. In RE-LY, there was a significantly higher risk of gastrointestinal bleeding with dabigatran 150 mg bid compared with warfarin (Connolly et al., 2009). No statistical significant difference was seen when using the 110 mg BID dabigatran dose. Mild to moderate dyspepsia related to dabigatran was also reported. The side effect tended to be transient, and may be managed by giving dabigatran with a large glass of water, with food, or a proton-pump inhibitor (PPI; Bytzer et al., 2012; Hoffman and Galle, 2013).

### Prevention of deep venous thrombosis

Dabigatran has been studied in more than 10,000 patients in four phase III trials of deep venous thrombosis (DVT) and pulmonary embolism (PE) prophylaxis in major orthopedic surgery and demonstrated a good overall safety profile in this setting (Fuji et al., 2010; Dahl et al., 2012; Eriksson et al., 2012a,b). In total knee replacement (TKR), when compared to enoxaparin 30 mg twice daily (BID), dabigatran etexilate at 150 and 220 mg once daily (OAD) was inferior due to a greater number of distal thromboses (Ginsberg et al., 2009). In studies comparing dabigatran to 40 mg of enoxaparin, equivalence was shown for both thrombosis prevention and bleeding in total hip replacement (THR) and TKR (Eriksson et al., 2007a,b). The THR results were reproduced in the RE-NOVATE II trial and dabigatran additionally demonstrated superior reduction of major VTE (including DVT and PE) with a same rate of major bleeding (Eriksson et al., 2011a). A pooled analysis of the THR trials demonstrated its superiority in the prevention of major VTE (Friedman et al., 2010; Eriksson et al., 2011b). In the countries where dabigatran etexilate is approved for VTE prophylaxis it is recommended at a dose of 220 or 150 mg OAD for patients with renal impairment or taking a Pgp inhibitor.

### Therapy of venous thromboembolism

In the treatment of VTE 150 mg BID dabigatran etexilate was compared to standard warfarin treatment (Schulman et al., 2009). In the acute treatment setting, all patients received heparin or LMWH for a median of 9 days prior to treatment with an oral anticoagulant. Dabigatran demonstrated the same efficacy in thrombosis prevention with significantly less minor bleeding and similar major bleeding. The extension studies RE-MEDY (vs. warfarin) and RE-SONATE (vs. placebo) showed similar efficacy and improved bleeding profile compared to warfarin, and a 92% relative risk reduction of recurrent VTE with elevated clinically relevant non-major bleeding but no statistical difference in major bleeding compared to placebo (Schulman et al., 2011a,b). The RE-SONATE study showed for the first time significant efficacy of an oral anticoagulant in the prevention of thromboembolic events with a good safety profile. Acute treatment and secondary prevention of VTE have not yet been registered.

### Stroke prevention in patients with atrial fibrillation

In the RE-LY study, 18,113 patients with atrial fibrillation (AF) and at least one risk factor for stroke were randomized between warfarin, 110 or 150 mg BID dabigatran etexilate (Connolly et al., 2009). The RE-LY trial was the only phase III study in the field of NOACs carrying enough power to evaluate two different doses and define the most appropriate patient population for a specific dose. The 150 mg BID dose showed superior efficacy in stroke prevention and systemic embolism with similar major bleeding rate compared to well controlled warfarin. The 110 mg BID dose had a significantly lower major bleeding rate and the same efficacy as warfarin. Both doses showed a significant (up to 70%) reduction of intracranial hemorrhage and a reduction in life-threatening bleeding. There was a non-significant 12% mortality benefit (*p* = 0.051) with the 150 mg BID dose (Connolly et al., 2010).

These results were consistent across all patient groups, including those with a prior history of stroke or transient ischemic attack (Diener et al., 2010), patients previously on warfarin, or those new to oral anticoagulation (Ezekowitz et al., 2010). Interestingly the effects seen under both doses were independent from gender, weight, ethnicity, renal impairment grade, CHADS_2_ scores, concomitant diseases (e.g., hypertension, diabetes, heart failure), and treatments (Connolly et al., 2009, 2010; Eikelboom et al., 2011). A RE-LY subgroup analysis has indicated an interaction between age and bleeding endpoints, with dabigatran 150 mg BID efficacy persisting for all age groups while dabigatran 110 mg BID in patients above 80 years improves safety profile (Eikelboom et al., 2011).

For stroke prevention, the FDA recommends a lower therapeutic dose (75 mg BID) in patients with severe renal impairment [creatinine clearance (CrCl) 15–30 ml/min] and no use in patients with CrCl under 15 ml/min. In Europe two doses (150, 110 mg BID) exist which enables tailored dosing and is contraindicated in patients with a CrCl below 30 ml/min.

### Prevention of recurrent myocardial infarction in patients with acute coronary syndrome

In the RE-DEEM phase II dose identification study, dabigatran plus dual antiplatelet therapy showed an expected dose-dependent increase in bleeding and significantly reduced coagulation activity (Oldgren et al., 2011). In the 150 and 110 mg BID dose groups, the composite of cardiovascular death, non-fatal myocardial infarction or hemorrhagic stroke or cardiovascular death or all-cause death alone were numerically lower compared to placebo (dual antiplatelet therapy). The safety profile in this post-ACS population was acceptable and a RE-LY subanalysis study found that concomitant use of antiplatelets led to an expected doubling of bleeding rates in both dabigatran and warfarin treatment groups (Connolly et al., 2009).

### Mechanical heart valves

Thromboprophylaxis after mechanical heart valve implantation is required for the lifetime of the patient, thus the concept of a DTI as a potential therapy was tested preclinically. *In vitro*, human blood anticoagulated with dabigatran circulating over a mechanical heart valve in a closed circuit effectively prevented fibrin deposition and thrombus formation compared to control (Maegdefessel et al., 2010). In an experimental swine model with a heart valve grafted onto the descending aorta, dabigatran administered post implantation for 30 days vs. LMWH significantly reduced valve thrombus and platelet deposition (McKellar et al., 2011). In a separate study, swine underwent mitral valve replacement and received dabigatran or warfarin for 90 days (Schomburg et al., 2012). Valve thrombus was observed in all groups; however there was decreased incidence of bleeding and a significant mortality benefit in the dabigatran-treated group vs. warfarin. Dabigatran’s effects in the setting of mechanical valve replacement was investigated in the dose-finding RE-ALIGN phase II study (Van de Werf et al., 2012), however the study was discontinued because interim analysis showed an increased incidence of thromboembolic events compared to warfarin. Therefore dabigatran is contraindicated in patients with mechanical prosthetic valves (FDA Drug Safety Communication, 2012).

## Summary

Based on a NAPAP-thrombin X-ray crystal structure a new class of thrombin inhibitors was designed, with the potency of these compounds optimized over several iterative steps. One inhibitor, dabigatran, demonstrated very strong *in vivo* activity, however due to its highly polar nature, oral absorption was insufficient. Several prodrugs were synthesized from which dabigatran etexilate emerged as a strong clinical candidate.

Preclinical pharmacological investigations illustrated that dabigatran is an effective antithrombotic agent in both venous and arterial models. In an unprecedented clinical trial program that included ∼38,000 patients, dabigatran was shown to be a highly effective anticoagulant with a good safety profile. Dabigatran is as effective as enoxaparin in the prevention of venous thrombosis after orthopedic surgery, and as effective as warfarin in the therapy of acute thrombosis or secondary prevention of VTE with no increased risk of bleeding. In stroke prevention in atrial fibrillation, it was more effective than well controlled warfarin with remarkable decreased risk of intracranial hemorrhage. Furthermore, since thrombin has been shown to play a central role in many disease processes, dabigatran is being investigated in additional biochemical pathways for potential beneficial effects in preclinical translational and disease models.

More than 50 years after the introduction of warfarin, the development of dabigatran can be seen as a breakthrough achievement for the treatment of thromboembolic disease including the prevention of catastrophic strokes in patients.

## Conflict of Interest Statement

All authors are employees of Boehringer Ingelheim GmbH.
